# Development of a Pacific oyster (*Crassostrea gigas*) 31,918-feature microarray: identification of reference genes and tissue-enriched expression patterns

**DOI:** 10.1186/1471-2164-12-468

**Published:** 2011-09-27

**Authors:** Nolwenn M Dheilly, Christophe Lelong, Arnaud Huvet, Pascal Favrel

**Affiliations:** 1 Université de Caen Basse-Normandie, UMR M100 "Physiologie et Ecophysiologie des Mollusques Marins", IBFA, IFR ICORE 146, Esplanade de la Paix, 14032 Caen Cedex, France; 2 Ifremer, UMR M100 "Physiologie et Ecophysiologie des Mollusques Marins", Technopole Brest Iroise, 29290 Plouzané, France

## Abstract

**Background:**

Research using the Pacific oyster *Crassostrea gigas *as a model organism has experienced rapid growth in recent years due to the development of high-throughput molecular technologies. As many as 56,268 EST sequences have been sequenced to date, representing a genome-wide resource that can be used for transcriptomic investigations.

**Results:**

In this paper, we developed a Pacific oyster microarray containing oligonucleotides representing 31,918 transcribed sequences selected from the publicly accessible GigasDatabase. This newly designed microarray was used to study the transcriptome of male and female gonads, mantle, gills, posterior adductor muscle, visceral ganglia, hemocytes, labial palps and digestive gland. Statistical analyses identified genes differentially expressed among tissues and clusters of tissue-enriched genes. These genes reflect major tissue-specific functions at the molecular level, such as tissue formation in the mantle, filtering in the gills and labial palps, and reproduction in the gonads. Hierarchical clustering predicted the involvement of unannotated genes in specific functional pathways such as the insulin/NPY pathway, an important pathway under study in our model species. Microarray data also accurately identified reference genes whose mRNA level appeared stable across all the analyzed tissues. *Adp-ribosylation factor 1 *(*arf1*) appeared to be the most robust reference for normalizing gene expression data across different tissues and is therefore proposed as a relevant reference gene for further gene expression analysis in the Pacific oyster.

**Conclusions:**

This study provides a new transcriptomic tool for studies of oyster biology, which will help in the annotation of its genome and which identifies candidate reference genes for gene expression analysis.

## Background

The Pacific oyster *Crassostrea gigas*, is one of the world's most economically important bivalves (4.2 million metrics tons annual production, worth $ 3.5 billion) [1]. It is also one of the best characterized models for biochemical, molecular and genetics studies among the Lophotrochozoa. *Crassostrea gigas*, and other oyster species, play an important role in estuarine and marine coastal habitats, where increasing human activity is causing environmental degradation [2]. In these ecosystems, Pacific oysters suffer summer mortalities due to increased stresses and disease outbreaks [3]. At the other extreme, *C. gigas *can become invasive in new habitats (e.g. in northern Europe) [4]. The scientific rationale of obtaining a wide variety of sequences and developing genomic tools for *C. gigas *is to take advantage of its membership of the Lophotrochozoa, an understudied clade of bilateria. Its study will contribute to knowledge in the fields of functional, comparative and evolutionary genomics by throwing new light on genome function and diversity [5-9], the evolution of sexuality [10-13] or immunity [14-16]. In the context of environmental genomics, oysters might also prove to be an attractive model organism for understanding population responses to environmental stresses and adaptation through genetic change, as well as for deciphering the physiological bases of complex traits (growth, reproduction, survival) [17-20].

In July 2010, 56,268 EST sequences were compiled in the publicly accessible GigasDatabase version 6 housed at Sigenae (http://public-contigbrowser.sigenae.org:9090/Crassostrea_gigas/index.html) [21]. This genomic resource allows the development of genome wide microarrays for screening physiological traits and responses to the environment. DNA microarray technology is a high-throughput method for measuring the expression levels of thousands of genes simultaneously. This approach has been applied extensively to establish gene expression patterns in many organisms, including yeast, worm, human, fruit fly and rice [22-26]. Investigators have already analyzed gene expression in *Crassostrea virginica *and *Crassostrea gigas *using cDNA microarrays constructed from 6,780 [27] and 9,058 [21] genes. These microarrays were used to describe gene expression patterns in response to heat stress [28] and hypoxia [29], and to compare oysters that were resistant or susceptible to summer mortalities [21]. Since these studies were done, a large database of ESTs has become available, providing the information necessary to design a more comprehensive microarray suitable for characterizing genome-wide transcriptome profiles in *Crassostrea gigas*.

Here, for the first time, we describe the design of an oligo-microarray covering a total of 31,918 contig sequences, and its application to monitoring gene expression profiles in multiple tissues of adult oysters. In addition to the first obvious advantage offered by this new microarray, which is the significant increase in the number of contigs per array, this Agilent oligonucleotide microarray uses shorter probes (60 mer instead of an average mean of 413 bp in *C. gigas *cDNA arrays), resulting in higher density arrays and cheaper manufacture. In addition, with respect to cDNA arrays, oligonucleotide microarrays are reported to provide higher sensitivity and reproducibility [30]. This work aims to survey variation in gene expression across multiple oyster tissue types, to provide experimental evidence for gene function assignments, and to identify gene clusters related to tissue-specific processes. Knowledge of tissue-specific expression is a prerequisite to any studies of organism development, normal functioning and response to injury and disease. Furthermore, the comparison of significantly different transcriptomes allowed the identification of genes with stable expression across tissues. Quantification of gene expression has become a crucial step to investigating any molecular mechanism or physiological process in oysters. However, the quality of normalized quantitative RT-PCR data is dependent on the accuracy of the normalizer itself. To avoid misinterpretation of the differential expression profile of a target gene, it is therefore necessary to identify reliable reference genes and test their stable expression across the different experimental conditions tested. Because microarray screening simultaneously unravels the expression of a huge number of genes, it offers the opportunity to discover genes with transcriptional stability that can serve as pertinent internal reference standards for future quantitative RT-PCR studies on oysters.

The present genome-wide analysis of gene expression in *C. gigas *tissues does not only establish lists of genes expressed in the surveyed tissues, but also provides evidence for the level of expression of these genes in different tissues. Importantly, this approach may provide clues for elucidating the functions of genes underlying specific processes and identify candidate genes predicted to regulate traits of interest. This argument is particularly relevant for the unannotated ESTs, for which such a survey can provide clues to resolving the orphan status.

## Results and discussion

### Robustness of *C. gigas *microarray data

We designed a microarray using 31,918 oligonucleotide 60-mer probes representing the presently known genes in the Pacific oyster *Crassostrea gigas *(GigasDatabase, version 6). Of the 31,918 probes printed onto the array, 20,170 (63.2%) corresponded to annotated contigs in the GigasDatabase. This microarray is intended for use in studies of oyster transcriptomes at the whole-genome level. In our study, the microarray was used to analyze the transcriptome of 9 tissues using one-channel hybridization (Cy3). The custom microarray design and the raw and normalized data is available from the Gene Expression Omnibus (GSE26265; http://www.ncbi.nlm.nih.gov/geo/query/acc.cgi?acc=GSE26265).

The homogeneous hybridization of the arrays was first estimated by comparing the expression profiles of duplicated probes. Thirty-four probe pairs were printed onto the arrays (Additional file 1). Expression values were highly reproducible with a mean R^2 ^of 95.7% (Additional file 1). An R^2 ^of 98.4% was obtained by removing data points with expression values below background levels.

**Figure 1:**
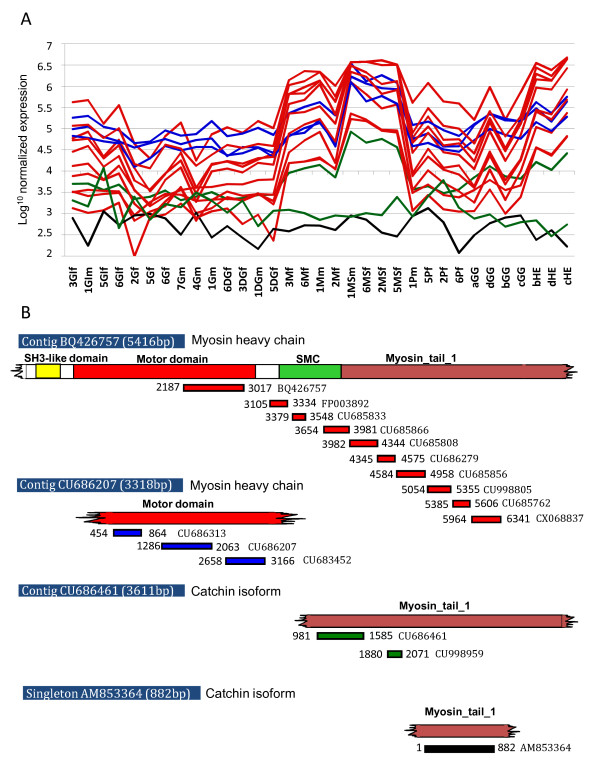
**Expression profiles of contigs similar to P24733, the myosin heavy chain of striated muscle in GigasDatabase version 6 (A) and the new alignement of these contigs in GigasDatabase version 8 (B)**. A. Fourteen probes similar to P24733 (Myosin Heavy Chain from *Argopecten irradians*) show the same profiles of transcript abundance (line) and appear more abundant in mantle (M), Muscle (MS) and Hemocytes (HE) than in other tissues, and two probes did not show significant variation of signal intensity among tissue samples (dots). For each biological sample, numbers identify the individuals (individuals 1 to 8), letters identify the tissues (GI: Gills; G: gonad; DG: Digestive gland; M: Mantle; MS: Adductor muscle; P: Labial palps; GG: Visceral ganglia; HE: Hemocytes), followed by m for males (individuals 1, 7 and 8) and f for females (individuals 2, 3, 5 and 6). a, b, c and d correspond to pools of 6 individuals necessary to extract a sufficient amount of RNA for both visceral ganglia and hemocyte samples. B. The 16 contigs similar to P24733 have been re-assembled into 3 contigs [BQ426757; CU686461; CU686207] and one singleton [AM853364] in the most recent assembly of *C. gigas *transcriptome (GigasDatabase, version 8, February 2011).

Next, to estimate the robustness of the microarray data, we compared the expression data obtained from different probes with identical annotation. Indeed, the oyster transcriptome is currently not completely sequenced and assembled and, therefore, some non full-length mRNAs are represented by numerous oligonucleotide probes printed on the array. This redundancy was used to confirm inter- and intra-array reproducibility. The consistency of the expression profiles obtained from different probes expected to come from the same mRNA confirmed the reproducibility of the data. For instance, among the 16 contigs originally annotated as similar to the myosin heavy chain of striated muscle from *Aequipecten irradians *(also known as *Argopecten irradians*) [GenBank: P24733] (GigasDatabase, version 6), 14 showed extremely similar expression profiles (Mean R^2^= 97.94%) (Figure 1A). The two contigs with different expression profiles showed very low gene expression values, close to or below background level, suggesting a low probe efficiency or a low expression in the different tissues. The most recent assembly of the *C. gigas *sequences (version 8, March 2011) constituted three contigs and one singleton from these 16 contigs (Figure 1B). Two contigs (BQ426757 and CU686207) encode two different myosin heavy chain isoforms (83% nucleotide identity). Contig CU686461 and singleton AM853364 encode two different isoforms of catchin transcript (86% nucleotide identity). Catchin is a protein present in molluscan catch muscles, which is produced by alternative splicing of myosin heavy chain [31,32]. In our dataset, myosin heavy chain and catchin present very similar expression profiles across tissues and a higher expression in catch muscle (Figure 1A).

Such strong profile consistency was frequently observed in our dataset such as the 3 sequences that matched the uncharacterized protein ZK643.6 (*Caenorhabditis elegans*) [GenBank: P30652]. Thus, our microarray data may prove useful in providing additional clues suggesting which contigs could constitute a single gene. The genes represented by multiple contigs can also be used to assess the reproducibility of the data and the homogeneity of the hybridization in future analysis. In Additional file 2 we provide a list of 200 putative genes significantly differentially expressed among tissues (ANOVA p < 0.05) and represented by 2 to 11 probes on the array. Over all contig pairs, we obtained a strong correlation of expression profiles (Mean R^2 ^= 83.2%).

Quantitative RT-PCR was performed for four selected candidate reference genes and six tissue-enriched genes (primers listed in Additional file 3). The patterns of transcript abundance detected for these genes in the array and in quantitative RT-PCR showed extremely similar profiles (mean R^2 ^= 85%) (Additional file 4). The correlation increased to 93% when we removed the hemocyte-enriched gene (BQ426482), which presented the lowest measured correlation between microarray and quantitative RT-PCR data (63%). Quantitative RT-PCR is commonly used as a validation tool for confirming gene expression results obtained from microarray analysis; however, microarray and quantitative RT-PCR data often disagree [33]. Using multiple probes corresponding to the same contig and comparing their resulting expression profiles may thus be a more accurate way of individually assessing microarray results and oligonucleotide specificity. A strong correlation between multiple probes corresponding to the same gene, as obtained in the present work, reflects high microarray quality.

### Prevalent gene expression patterns

**Figure 2:**
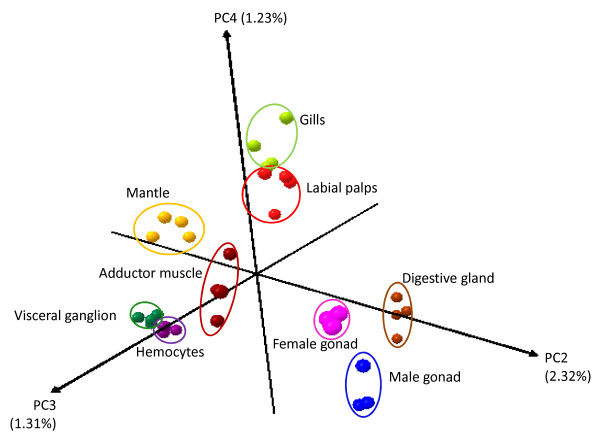
**3D score plots using principal components (PC) identified by principal components analysis (PCA)**. Cumulative data of all 33 tissue samples for all oyster genes printed onto the array. Different individual samples from the same tissue type grouped together, and most tissue types were clearly different from each other.

We applied principal component analysis (PCA) to the whole dataset. The PCA of all genes in the surveyed tissues showed that datasets acquired from the same tissue type grouped together, indicating that there is a strong homogeneity in the gene expression pattern in each tissue type (Figure 2). Furthermore, tissues with similar functions had a lower scattering, as observed for the transcriptomes of gills and labial palps. Indeed, gills and labial palps are two pallial organs, both secreting mucus and possessing similar structures involved in particle selection, which is an essential part of bivalve filter-feeding.

It was not possible to extract a sufficient amount of total RNA for microarray hybridization from a single visceral ganglion or from hemolymph of a single individual. Therefore, we randomly pooled samples from 6 individuals. Pooling results in biological averaging for most genes. Consequently, the variation among samples in these two tissue categories is much lower than the variation among individuals for other tissues (Figure 2). However, Kendziorski et al. [34] demonstated that the construction of a large number of pools enables the biological variation to be properly assessed and comparative studies to be made.

**Figure 3:**
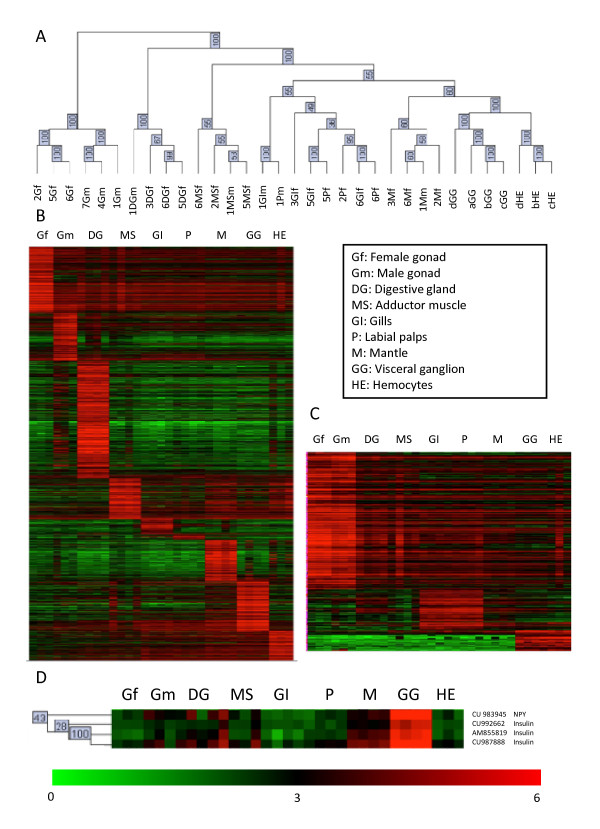
**Unsupervised hierarchical clustering of the gene expression profiles**. (A) Classification of the 33 biological samples using unsupervised hierarchical clustering of the gene expression profiles (31,918 contigs) employing Pearson's correlation. Numbers along the branches indicate bootstrap values after 100 iterations. Labial palp and gill tissue samples from the same individuals grouped together (1Pm and 1Bm; 5Pf and 5Bf; 6Pf and 6Bf). (B) Heat map of selected genes specifically overexpressed in different tissue types. Individual columns represent individual biological samples. The number of genes enriched in each tissue is indicated in Table 1. (C) Heat map of selected genes specifically over-expressed in two tissue types. (D) Expression of annotated and non-annotated genes potentially involved in regulating food-intake and energy balance. The cluster of genes was obtained using hierarchical clustering with Pearson's correlation on the tissue-enriched genes. For each biological sample, numbers identify the individuals (individuals 1 to 8) and letters show the pools of 6 individuals (a, b, c and d), upper letters identify the tissues (G: Gonad; GI: Gills; P: Labial palps; M: Mantle; MS: Adductor muscle; DG: Digestive gland; GG: visceral ganglia; HE: Hemocytes), followed by m for males (individuals 1, 7 and 8) and f for females (individuals 2, 3, 5 and 6).

To further explore relationships among tissue samples, we performed a hierarchical clustering analysis on all the genes printed on the array. The results were extremely similar to those of the PCA analysis (Figure 3A). Gonads grouped together on a single branch, separated from all other tissues with 100% bootstrap support, demonstrating that the transcriptome of gonads (regardless of gender) is significantly different from the transcriptome of somatic tissues. Hierarchical clustering then further separated individual gonad samples according to their gender. The data also showed that the digestive gland and adductor muscle clearly had different transcriptomes, with 100% bootstrap support. Mantle tissue clustered apart from visceral ganglion and hemocyte samples with 60% bootstrap support. However, the hierarchical cluster analysis could not differentiate between labial palps and gills. Interestingly, labial palps and gills from the same individual always clustered together (1Pm with 1GIm, 5Pf with 5GIf and 6Pf with 6GIf) on the hierarchical clustering tree, once again demonstrating the strong similarities between these two tissues (Figure 3). Further analysis revealed that these organs co-expressed genes in relation with their common biological functions (see below).

### Tissue-enriched gene expression

In the absence of a functional assay, tissue-specific gene expression features have often been viewed as indicators of tissue-specific function. In order to identify genes with differential tissue-enriched expression patterns, we initially performed a one-way ANOVA with a p-value threshold < 0.01, adjusted with Bonferroni's correction. A total of 7,586 contigs were differentially expressed between tissues (Table 1). These differentially expressed contigs were further filtered to determine whether they were overexpressed in a particular tissue. We required that the mean of the log_10 _normalized expression level of the replicates in a given tissue was at least 1.1 fold higher than the means in each of the other tissues (see Methods). A 1.1 fold increase of log_10 _values is equivalent to a 1.26 fold increase of the raw data. By this process, tissue-enriched contigs were identified for all tissues analyzed (Figure 3B).

**Table 1:** Distribution of tissue-enriched contigs

Tissue	number of genes	Annotated in GigasDatabase	
ANOVA significant	7,586	3,417	(45%)

Female Gonad	268	119	(44%)

Male Gonad	195	95	(49%)

Gills	61	15	(25%)

Digestive gland	471	240	(51%)

Mantle	165	45	(28%)

Adductor Muscle	161	69	(43%)

Visceral ganglia	199	86	(43%)

Labial Palps	23	10	(44%)

Hemolymph	122	47	(39%)

The number of tissue-enriched contigs varied substantially, ranging from 23 contigs in labial palps to 471 in the digestive gland. The number of tissue-enriched contigs and the proportion of contigs annotated in the GigasDatabase are shown in Table 1. Figure 3B shows the heat map of the tissue-enriched contigs, and the complete list with their descriptions are given in Additional file 5. The highest percentage of contigs with annotation was obtained for genes specifically expressed in the digestive gland (51%). This is almost certainly because digestive processes are ancient and highly conserved. In digestive glands, many enzymes are widely distributed and conserved over a large range of species. For example, alpha-amylase, a key carbohydrase enzyme for starch digestion has been characterized from bacteria, fungi, plants and animals [35] and high sequence identity was observed for the domains responsible for its enzymatic activity [36]. It is also possible that there were qualitative differences in the original sequencing of mRNAs from the different tissues, which would result in poor BLAST matches. The constant updating of GigasDatabase will allow improvement of the sequence descriptions.

The annotated tissue-enriched contigs showed a strong relationship with the physiological functions of the corresponding tissues (Additional file 5). For example, numerous genes involved in digestion were highly expressed in the digestive gland (*clh2, chia, plb1, pnlip, muc2, prss7, amy1, xynx, cpa1, lct *and *cst3*), whereas genes involved in tissue growth and development of hard tissues were highly expressed in the mantle (*kcp, pxdn, chi3l4, fam20c, tnxb *and *col6a3*). Similarly, the visceral ganglia expressed numerous genes related to its function in the regulation of neurotransmitter release and hormone secretion (*syt7, mrp, syt12, at5g38780, npy, cex-1, sspo, acr-2, takr86C, cmd-1, pcsk2, nAcRalpha, dbh, unc-64 *and *pin*). Interestingly, in addition to well-characterized neuropeptide encoding genes (*neuropeptide Y *[CU983945], *PRQFV-amide-related peptides *[CU986397], *orcokinin-like peptides *[AM854447] and *pedal peptide *[CU988369, CX069323]), some of the non-annotated ganglia-specific contigs were found to encode the precursors of putative novel neuropeptides [CU995163, CU992964, AM868259] that the BLAST tool did not identify, probably due to limited sequence conservation between peptides or their precursors [37]. These putative novel neuropeptides displayed the conventional dibasic (KR, RR) cleavage sites for prohormone convertases and potentially downstream GKR sequencing serving as combined amidation and proteolytic signals.

The annotation of a high proportion of the genes printed onto this array facilitates the exploration of specific biological processes and the identification of genes potentially involved in the same pathways. For example, of the genes specifically expressed in the visceral ganglia, we found that neuropeptide Y [CU983945] and insulin [CU992662, AM855819 and CU987888] clustered closely (Figure 3D). NPY and insulin are involved in the processes of the food intake regulation, allocation of energy to growth, reproduction and basal metabolism in both vertebrates and invertebrates [38-40]. Such regulatory molecules deserve particular attention, especially in the context of the summer mortality syndrome in which reproductive effort plays a crucial role through its effects on the capacity of oysters to survive [13,15,41]. Overall, genes without significant annotation were specifically attributed to a tissue based on their expression pattern, which can provide valuable help towards the assignment of gene function. Indeed, even if gene function is primarily assessed on the basis of altered phenotypes associated with gene disruption, knowledge of spatio-temporal expression patterns of a given gene represents valuable information for assigning a putative function.

Some functional clusters also showed similarities between tissues, such as male and female gonads, both of which expressed significantly higher levels of 341 genes than all other tissues, or labial palps and gills, which showed significantly higher expression of a common set of 101 genes. Hemocytes and visceral ganglia shared 54 overexpressed genes (Figure 3C). The complete list of dual tissue-enriched contigs and their descriptions are provided in additional file 6. As expected, male and female gonads showed the highest expression levels of all cell cycle, mitosis and meiosis regulation genes, including cell cycle checkpoint (*rad1, fbxo43, mad2a, clspn *and *cdkn3*), control of replication (*mcm3, -2 and -7, rfc3, -4 and -5, Gins1, -2 *and *-3*) and DNA repair genes (*mre11, xrcc1 and -3, rad54b, -51b, -50 and -21, msh6, lig1 *and *top2b*). Gills and labial palps, the main feeding organs of oysters, expressed 37 genes potentially involved in epithelia morphogenesis (*mark2, plscr2 *and *pfn4*), cilia movement (*vil1, cam *and *srI*) and in detoxification and defense mechanisms (*gst, ces3, gpx1, adamts18 *and *pox1*). The visceral ganglia and hemocytes mainly shared a high expression in topoisomerase I (6 genes), two G protein-coupled receptors (*nmur1 *and *agtr1*) whose vertebrate orthologues bind neuropeptides mediating blood pressure regulation and stress response [42,43], and a set of defense-related genes (*big defensin, pxn-1, hsp27 *and *piap*) whose expression in the central nervous system suggests a concerted activity with hemocytes in defense and stress response [14,44].

### Reference genes

The term "housekeeping gene" is generally applied to genes ubiquitously expressed at the same level in all tissues. These constitute the basal transcriptome for the maintenance of basic cellular functions. Their uniform expression means they can be used as reference genes for quantitative RT-PCR [45]. Thus, we investigated the stability of gene expression levels to identify appropriate reference genes. To allow for comparisons of means and standard deviations, logarithmic normalized data were centered on their mean (fixed at 4). A candidate reference gene was defined as a gene that was not differentially expressed when tested by ANOVA (p-value < 0.05) and which had a coefficient of variation (CVarray, the ratio of the standard deviation to the mean) of < 5%, a maximum fold change (MFC, the ratio of the maximum and minimum values observed within the dataset) of < 1.1, and a mean expression level higher than the average expression on the array, i.e., > 4. One hundred and twenty-five unique genes were selected as reference candidate genes and ranked in order of increasing CVarray (Additional file 7). Among these, the 49 annotated reference gene candidates are shown in table 2.

**Table 2:** The 49 annotated candidate reference genes ranked in order of their coefficient of variation (spot: location on microarray, ID genbank: accession number, stdev: standard deviation, description: best BLAST hit description as described in GigasDatabase, e value: best BLAST hit e-value, CV: coefficient of variation, max: maximum value, min: minimum value, MFC: maximum fold change, p value: one-way ANOVA P > 0.05)

RANK	spot	ID genebank	description (GigasDatabase)	e value	MEAN	stdev	CV	max	min	MFC	p value
2	24202	BQ426480	(sp:Q66KU2) Protein transport protein Sec61 subunit gamma OS = Xenopus laevis GN = sec61g PE = 3 SV = 1	7E-20	6.16	0.07	0.012	6.32	6.01	1.05	0.28

4	8432	BQ427048	(sp:P61210) ADP-ribosylation factor 1 OS = Locusta migratoria GN = ARF1 PE = 2 SV = 2	2E-92	5.82	0.08	0.014	5.98	5.61	1.07	0.13

5	4341	FP005681	(sp:Q80TM9) Nischarin OS = Mus musculus GN = Nisch PE = 1 SV = 2	2E-17	5.28	0.07	0.014	5.42	5.14	1.05	0.18

7	9190	AM856720	(sp:Q3SXD3) HD domain-containing protein 2 OS = Mus musculus GN = Hddc2 PE = 2 SV = 1	5E-59	4.04	0.06	0.014	4.16	3.93	1.06	0.39

12	4953	FP007578	(sp:P55260) Annexin A4 OS = Rattus norvegicus GN = Anxa4 PE = 1 SV = 3	3E-24	5.80	0.09	0.015	5.99	5.64	1.06	0.05

17	3774	EW777430	(sp:Q80ZA5) Sodium-driven chloride bicarbonate exchanger OS = Rattus norvegicus GN = Slc4a10 PE = 2 SV = 1	2E-87	5.57	0.09	0.016	5.78	5.39	1.07	0.22

18	16231	AM867010	(sp:Q6PDY2) 2-aminoethanethiol dioxygenase OS = Mus musculus GN = Ado PE = 1 SV = 2	3E-24	4.68	0.07	0.016	4.84	4.55	1.06	0.37

23	24189	AM862676	(sp:P49756) RNA-binding protein 25 OS = Homo sapiens GN = RBM25 PE = 1 SV = 3	8E-38	5.17	0.08	0.016	5.31	4.97	1.07	0.11

25	21853	AM867442	(sp:Q9CSV6) Vesicle transport protein SFT2C OS = Mus musculus GN = Sft2d3 PE = 2 SV = 2	8E-17	4.92	0.08	0.016	5.08	4.74	1.07	0.08

29	21268	FP007036	(sp:Q5RE10) Protein TSSC1 OS = Pongo abelii GN = TSSC1 PE = 2 SV = 1	7E-93	5.26	0.09	0.017	5.52	5.09	1.08	0.06

31	2316	FP004268	Branchiostoma floridae hypothetical protein (BRAFLDRAFT_123626) mRNA, complete cds	6E-11	5.72	0.10	0.017	5.96	5.46	1.09	0.18

35	27321	CU998396	(sp:Q5U5C5) U4/U6 small nuclear ribonucleoprotein Prp31 OS = Xenopus laevis GN = prpf31 PE = 2 SV = 1	2E-42	5.49	0.10	0.018	5.70	5.32	1.07	0.09

36	14581	CU682778	(sp:Q96ME1) F-box/LRR-repeat protein 18 OS = Homo sapiens GN = FBXL18 PE = 2 SV = 2	4E-13	4.67	0.09	0.018	4.97	4.53	1.10	0.12

39	38790	FP007991	(sp:Q8IY22) C-Maf-inducing protein OS = Homo sapiens GN = CMIP PE = 1 SV = 2	2E-62	5.54	0.10	0.019	5.73	5.32	1.08	0.31

43	17127	CU994599	(sp:O16099) Maltase 2 OS = Drosophila virilis GN = Mav2 PE = 3 SV = 1	6E-15	6.04	0.11	0.019	6.29	5.84	1.08	0.08

44	38094	AM854363	(sp:P49790) Nuclear pore complex protein Nup153 OS = Homo sapiens GN = NUP153 PE = 1 SV = 2	1E-06	5.36	0.10	0.019	5.52	5.13	1.08	0.35

45	42336	AM867367	(sp:Q9V8K2) Exocyst complex component 3 OS = Drosophila melanogaster GN = sec6 PE = 1 SV = 2	2E-46	4.07	0.08	0.019	4.24	3.90	1.09	0.51

48	18292	AM857903	(sp:Q9HBH5) Retinol dehydrogenase 14 OS = Homo sapiens GN = RDH14 PE = 1 SV = 1	1E-52	4.86	0.09	0.019	5.06	4.69	1.08	0.98

50	23889	AM857691	(sp:Q53HI1) Protein unc-50 homolog OS = Homo sapiens GN = UNC50 PE = 1 SV = 2	1E-44	4.94	0.10	0.019	5.19	4.73	1.10	0.14

51	9261	CB617377	(sp:P35980) 60S ribosomal protein L18 OS = Mus musculus GN = Rpl18 PE = 2 SV = 3	3E-52	5.62	0.11	0.019	5.88	5.41	1.09	0.12

57	6325	AM854187	(sp:Q8BI36) JNK1/MAPK8-associated membrane protein OS = Mus musculus GN = Jkamp PE = 1 SV = 2	4E-54	4.06	0.08	0.020	4.23	3.90	1.08	0.97

59	26590	CU991951	(sp:O75592) Probable E3 ubiquitin-protein ligase MYCBP2 OS = Homo sapiens GN = MYCBP2 PE = 1 SV = 3	1E-93	5.48	0.11	0.020	5.70	5.24	1.09	0.06

63	14888	CU999965	(sp:Q99323) Myosin heavy chain, non-muscle OS = Drosophila melanogaster GN = zip PE = 1 SV = 2	4E-61	5.47	0.11	0.020	5.66	5.25	1.08	0.06

65	28114	AM865894	(sp:Q297U0) Death domain-containing adapter protein BG4 OS = Drosophila pseudoobscura pseudoobscura GN = BG4 PE = 3 SV = 2	9E-06	5.41	0.11	0.020	5.59	5.19	1.08	0.15

71	25525	FP001776	(sp:P38935) DNA-binding protein SMUBP-2 OS = Homo sapiens GN = IGHMBP2 PE = 1 SV = 2	3E-22	4.90	0.10	0.021	5.13	4.72	1.09	0.05

72	31850	AM859152	hypothetical protein [Leishmania infantum]	7E-06	4.51	0.09	0.021	4.65	4.27	1.09	0.08

73	11422	AM857565	(sp:O75689) Arf-GAP with dual PH domain-containing protein 1 OS = Homo sapiens GN = ADAP1 PE = 1 SV = 2	2E-90	5.78	0.12	0.021	6.04	5.51	1.10	0.21

74	9287	AM858936	(sp:O75970) Multiple PDZ domain protein OS = Homo sapiens GN = MPDZ PE = 1 SV = 1	2E-54	4.01	0.08	0.021	4.16	3.84	1.08	0.26

76	12345	FP002886	(sp:Q9UP83) Conserved oligomeric Golgi complex subunit 5 OS = Homo sapiens GN = COG5 PE = 1 SV = 2	1E-40	5.12	0.11	0.021	5.46	4.98	1.10	0.15

77	16551	AM854998	(sp:P34384) Uncharacterized protein F02A9.4b OS = Caenorhabditis elegans GN = F02A9.4 PE = 2 SV = 3	6E-16	4.88	0.10	0.021	5.13	4.71	1.09	0.07

78	37299	AM854324	(sp:O14681) Etoposide-induced protein 2.4 homolog OS = Homo sapiens GN = EI24 PE = 1 SV = 4	3E-46	4.34	0.09	0.021	4.54	4.20	1.08	0.12

79	44361	AM867088	hypothetical protein LOC100216081 [Xenopus (Silurana) tropicalis]	8E-07	4.03	0.09	0.021	4.22	3.90	1.08	0.43

82	4930	CX069207	(sp:P24392) Peroxisome assembly factor 1 OS = Rattus norvegicus GN = Pxmp3 PE = 2 SV = 1	1E-14	4.24	0.09	0.022	4.42	4.03	1.10	0.49

83	23819	DW713831	(sp:Q8R107) PRELI domain-containing protein 1, mitochondrial OS = Mus musculus GN = Prelid1 PE = 2 SV = 1	5E-31	4.31	0.09	0.022	4.45	4.13	1.08	0.28

88	42190	CU999658	(sp:Q4R4P4) Fatty acid 2-hydroxylase OS = Macaca fascicularis GN = FA2H PE = 2 SV = 1	2E-37	4.93	0.11	0.022	5.09	4.69	1.09	0.13

90	41203	AM861170	(sp:O95562) Vesicle transport protein SFT2B OS = Homo sapiens GN = SFT2D2 PE = 1 SV = 1	2E-45	4.67	0.10	0.022	4.92	4.50	1.09	0.39

92	28026	FP003987	(sp:P28668) Bifunctional aminoacyl-tRNA synthetase OS = Drosophila melanogaster GN = Aats-glupro PE = 1 SV = 2	9E-26	5.45	0.12	0.023	5.76	5.24	1.10	0.34

96	28299	AM860951	(sp:Q63055) ADP-ribosylation factor-related protein 1 OS = Rattus norvegicus GN = Arfrp1 PE = 2 SV = 1	3E-61	4.58	0.10	0.023	4.77	4.34	1.10	0.42

99	1305	CU987227	(sp:O75533) Splicing factor 3B subunit 1 OS = Homo sapiens GN = SF3B1 PE = 1 SV = 3	5E-39	4.04	0.09	0.023	4.21	3.90	1.08	0.42

101	19889	FP003031	(sp:A1Z623) 15 kDa selenoprotein OS = Sus scrofa GN = SEP15 PE = 2 SV = 2	2E-27	5.58	0.13	0.023	5.86	5.34	1.10	0.08

102	31829	CX739641	(sp:P06603) Tubulin alpha-1 chain OS = Drosophila melanogaster GN = alphaTub84B PE = 2 SV = 1	1E-173	6.28	0.15	0.024	6.57	5.99	1.10	0.07

106	27419	FP004307	(sp:Q7KHA1) Phosphoglucomutase OS = Drosophila simulans GN = Pgm PE = 3 SV = 1	2E-08	4.40	0.11	0.024	4.61	4.23	1.09	0.26

109	9265	CB617357	(sp:P62925) Eukaryotic translation initiation factor 5A OS = Spodoptera frugiperda GN = eIF-5A PE = 2 SV = 1	9E-52	5.63	0.14	0.024	5.91	5.42	1.09	0.31

112	19642	AM859051	(sp:Q5RAK3) RING finger protein 180 OS = Pongo abelii GN = RNF180 PE = 2 SV = 1	2E-08	4.47	0.11	0.024	4.69	4.30	1.09	0.11

113	41799	AM855515	(sp:Q6GMK8) Mannose-1-phosphate guanyltransferase alpha-A OS = Danio rerio GN = gmppaa PE = 2 SV = 1	1E-119	4.62	0.11	0.024	4.82	4.40	1.10	0.24

121	12762	FP001461	(sp:Q9EPS3) D-glucuronyl C5-epimerase OS = Mus musculus GN = Glce PE = 1 SV = 1	1E-19	4.96	0.13	0.026	5.19	4.76	1.09	0.13

122	43827	FP002120	(sp:Q3SZV2) UPF0459 protein C19orf50 homolog OS = Bos taurus PE = 2 SV = 1	9E-21	4.66	0.12	0.026	4.86	4.42	1.10	0.09

124	30267	CX069057	(sp:Q5R9Z1) Vacuolar protein sorting-associated protein 29 OS = Pongo abelii GN = VPS29 PE = 2 SV = 1	5E-93	5.58	0.15	0.028	5.84	5.34	1.09	0.06

125	14686	AM862926	(sp:Q3TEA8) Heterochromatin protein 1-binding protein 3 OS = Mus musculus GN = Hp1bp3 PE = 1 SV = 1	4E-14	4.89	0.13	0.028	5.16	4.69	1.10	0.10

CV indicates the coefficient of variation and equals the standard deviation divided by the mean. MFC indicates the maximum fold change, i.e., the ratio of the maximum and minimum values observed within the dataset.

Remarkably, none of the 3 most commonly used reference genes in oyster (*actin, g3apdh *and *ef1α*) ranked within the top 125 identified candidate reference genes. *Actin *and *g3apdh *had p-values < 0.05 when tested by ANOVA and were not ranked (Table 3). *Ef1α *did not show any significant variation when tested by ANOVA but, similarly to *actin *and *g3apdh*, it had an MFC > 1.1. Even if the MFC had not been taken into account, *ef1α *would still have ranked only 256^st ^among the candidate genes (Table 3).

**Table 3:** Ranking of 3 commonly used reference genes for quantitative real time PCR experiments on oysters (spot: location on microarray, ID genbank: accession number, stdev: standard deviation, description: best BLAST hit description as described in GigasDatabase, CV: coefficient of variation, max: maximum value, min: minimum value, MFC: maximum fold change, p value: ANOVA 1 factor, P > 0.05, NR: Not Ranked)

RANK	spot	ID genbank	description (GigasDatabase)	MEAN	stdev	CV	max	min	MFC	p value
256	11936	BG467400	(sp:Q9YIC0) Elongation factor 1-alpha OS = Oryzias latipes GN = eef1a PE = 2 SV = 1	4.08	0.10	0.03	4.29	3.86	**1.11**	0.41

NR	10575	AJ544886	(sp:P56649) Glyceraldehyde-3-phosphate dehydrogenase OS = Panulirus versicolor PE = 1 SV = 1	6.17	0.20	0.03	6.57	5.79	**1.13**	**< 0.05**

NR	26871	AF026063	(sp:O17320) Actin OS = Crassostrea gigas PE = 2 SV = 1	6.41	0.16	0.02	6.67	6.01	**1.11**	**< 0.05**

CV indicates the coefficient of variation and equals the standard deviation divided by the mean. MFC indicates the maximum fold change, i.e., the ratio of the maximum and minimum values observed within the dataset.

**Figure 4:**
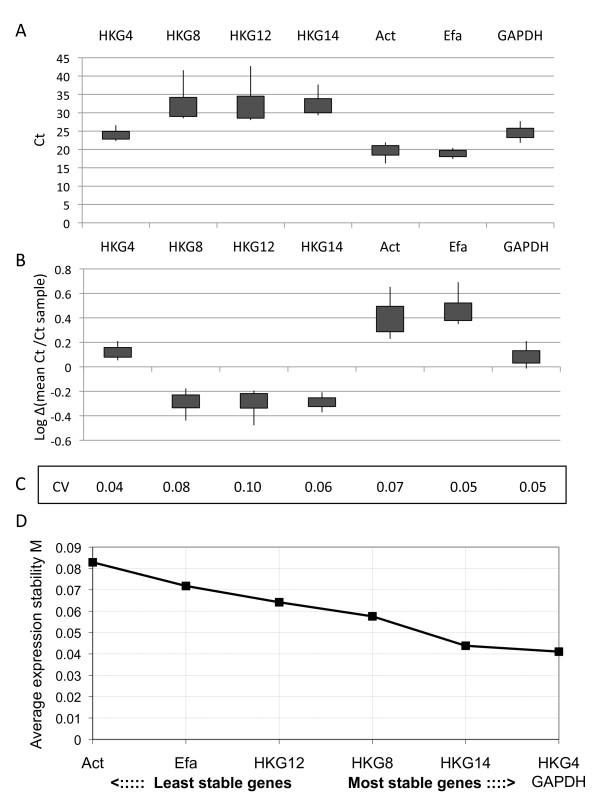
**Expression stability of reference genes**. Results of quantitative RT-PCR analysis are depicted as (A) Ct values measured or (B) Log (mean Ct/Ct sample) for each gene analyzed. Box plots graphically depict the minimum value, lower quartile, upper quartile and maximum value. (C) Coefficient of variation (CV; ratio of the standard deviation and the mean). (D) Average expression stability as calculated using geNorm. For details see Material and Methods. Genes are ordered according to increasing expression stability, identifying *hkg4 *and *g3apdh *as the best housekeeping genes for reference.

To demonstrate the importance of identifying appropriate reference genes, we designed primers for four of the 15 top-ranked novel reference genes (*hkg4, hkg8, hkg12 *and *hkg14*) and compared their mRNA levels estimated by quantitative RT-PCR with those of the three commonly used reference genes (*actin, ef1α *and *g3apdh*) in all tissue samples. To normalize data across samples, the mean of Ct values across all genes was calculated for each sample. The log values (mean Ct /Ct sample) were then compared for each gene. Box plots showing the minimum, maximum and mean value of Ct and log value (mean Ct/Ct sample) for each gene are shown in Figure 4A and 4B. In both datasets, *hkg4*, annotated as an *adp-ribosylation factor 1 *(*arf1*) in GigasDatabase, and *g3apdh *were the most stable genes, with coefficients of variation (CV; ratio of the standard deviation on the mean) of 0.04 for *hkg4 *and 0.05 for *g3apdh*. They also had the lowest CVs among all genes tested (Figure 4C). Applied to the raw data, Normfinder identified *g3apdh *as the best reference gene over the ensemble of the tissues, whereas it identified *hkg4 *as the best reference gene when considering normalized data. Using geNorm analysis on non-normalized expression levels, we determined the gene-stability expression measures (M) for the seven candidate reference genes. Again, *hkg4 *and *g3apdh *were the most stable reference genes, with an average expression stability value of 0.041 (Figure 4D). *Arf1 *has also been identified as a housekeeping gene in humans [46]. ADP-ribosylation factors are highly conserved 21 kDa GTPases involved in vesicular trafficking in all eukaryotes [47]. Glyceraldehyde-3-phosphate dehydrogenase (GAPDH) is one of the most commonly used housekeeping genes for comparisons of gene expression data, though variability of GAPDH expression between human tissue types was recently shown [48]. In *C. giga*s, *g3apdh *was found to be significantly more expressed in the adductor muscle (ANOVA p < 0.05) and should, thus, be avoided when studying gene expression across different tissues. However, its expression appeared stable within tissues suggesting that it may be suitable for other experimental designs. We therefore identified ADP ribosylation factor 1 (*arf1*) as being a more relevant reference gene for use in normalization of real time PCR for tissue-expression studies than those typically used. In addition, the list of the 125 potential reference genes will clearly help in the choice of references genes in further gene expression studies, but we still emphasize the need to experimentally validate reference genes for specific experimental designs [45].

## Conclusions

Research on gene function and oyster physiology using real time PCR and microarray is becoming increasingly important and widespread. Because of their economic importance, evolutionary position and use as a model organism in environmental genomics, more research is now being conducted on oyster species. A large EST project lead by French and US teams generated a genome-wide EST database, GigasDatabase, that is now freely available and housed on the Sigenae website. In the present study, the GigasDatabase was used to generate oligo-microarrays, providing the genomic tools necessary to undertake high-throughput studies of significantly important phenomena, such as summer mortalities in bivalves, host-pathogen interactions, reproduction and the effect of polyploidy on physiology of Pacific oysters. However, in order to make sense of gene expression data, we developed a 32 K microarray containing all known available contigs of *C. gigas*, thus aiming to represent the whole transcriptome. This study fulfilled 3 main goals. (1) It designed and presented a genome-wide oyster microarray that can now be employed in different gene expression studies in Pacific oysters (GSE 26265). The microarray is publicly available, following a request to the Agilent company and an authorization given by our team to share and use the oyster design. (2) It described the basic transcriptome of nine different tissues, providing important information for gene annotation and function prediction for genome sequencing. (3) It identified a large array of genes that can be used as reference genes to normalize gene expression data.

## Methods

### Animal sampling

Twenty-four adult oysters (3 years old) were sampled in July 2010 in Baie des Veys (Normandy, France). First, hemolymph was extracted from the adductor muscle using a 23-gauge needle attached to a 1-ml syringe, then gonad, gills, digestive gland, the posterior adductor muscle, labial palps, mantle and visceral ganglions were dissected. At this stage, pools of 6 individuals were randomly constituted for hemolymph and visceral ganglion samples. All samples were snap-frozen in liquid nitrogen, and stored at -80°C. Total RNA was extracted from individual tissues (3 female gonads, 3 male gonads, 4 gills, 4 digestive glands, 4 posterior adductor muscles, 4 labial palps, 4 mantles) or from pools of 6 individuals (4 pools of visceral ganglions and 3 pools of hemocytes) with Tri-Reagent kit (Sigma) and subsequently cleaned with Nucleospin RNA Clean-Up (Macherey Nagel) isolation columns. After the homogenisation of the N_2 _grinding powder in Tri-Reagent and the first centrifugation, the aqueous phase was extracted to directly isolate the total RNA on the column, as described by the manufacturer. Absence of trace genomic DNA was confirmed by gel electrophoresis. RNA concentrations were determined using a ND-1000 spectrophotometer (Nanodrop Technologies) at 260 nm, using the conversion factor 1 OD = 40 μg/ml RNA. RNA integrity was verified on an Agilent bioanalyzer using RNA 6000 Nano kits (Agilent Technologies), according to manufacturer's instructions, without consideration for the RNA Integrity Number (RIN) [49]. Indeed, in molluscan RNA, the co-migration of the 28S rRNA fragments with the 18S rRNA prevented us from using the RIN, thus only the absence of RNA degradation can be considered [50-52]. A detailed description of the validation of RNA integrity in oysters is provided in Additional file 8. Samples were stored at -80°C until use.

### Oligonucleotide microarray design and construction

The sequences used to design oligonucleotide probes were obtained from the publicly accessible GigasDatabase version 6, available on the Sigenae website (http://public-contigbrowser.sigenae.org:9090/Crassostrea_gigas/index.html) [21]. In July 2010, the final dataset contained 31,952 contigs assembled from 56,268 sequences selected from cDNA libraries derived from a wide variety of oyster tissues and developmental stages (Additional file 9). Contigs had previously been annotated with BLAST × and added to GigasDatabase [21]. From the resulting 31,952 contigs, 60-mer oligonucleotidic probes were designed using eArray software with the default parameters (Agilent Technologies). After repeat masking, low complexity filtering and similarity searches, 31,918 sequences contained exploitable probes. One probe per sequence was then printed onto a 4 × 44 K custom GE Microarray (Agilent Technologies). This automatic probe design generated 34 duplicated probes from highly similar sequences (Additional file 1). These probes were used to evaluate the reproducibility of hybridization (see results). Probes for negative controls were also printed onto the array *in situ *and used to test for low background before analysis (see Correction and Normalization). A new assembly (Gigas Database version 8) has been generated since the end of this study. The retrieval of sequences is possible using a Genbank name query, followed by .p.cg.8. All sequences used in this study are also available in Genbank and EMBL and are given in Additional file 9.

### Oligonucleotide microarray hybridization

#### RNA Amplification, labeling and hybridization

For microarray hybridizations, 200 ng of total RNA was indirectly labeled with Cy3 using the Low Input Quick Amp labeling kit (Agilent Technologies), according to the manufacturer's instructions. Qiagen RNeasy mini spin columns were used for purifying amplified RNA (aRNA) samples. After purification, aRNA amplification and dye incorporation rates were verified using a ND-1000 spectrophotometer (Nanodrop Technologies) and shown to lie between 200 and 500 ng/μL (aRNA concentration) and between 20 and 50 pmol/μg aRNA (dye incorporation).

Hybridization was performed using the Agilent Gene expression hybridization kit (5188-5242), as described by the manufacturer, with 1.65 μg of aRNA labeled with Cy3. Tissue samples were randomly hybridized onto 10 different slides, which were subsequently treated with Gene expression wash buffer solution (5188-5327; Agilent Technologies), and Stabilization and Drying solutions (5185-5979; Agilent Technologies). Finally, slides were scanned on an Agilent Technologies G2565AA Microarray Scanner system at 5 μm resolution, using default parameters.

#### Correction and Normalization

Feature extraction and data normalization were conducted with Agilent Feature Extraction software 6.1. (Agilent Technologies), using the default/recommended normalization method. Initial microarray testing revealed cross hybridization of *C. gigas *aRNA on the spiked-in probes. Thus Agilent spike-ins were not included in this study and are not recommended for future microarray analysis of *C. gigas*. The array contained 153 negative control spots with a mean gene expression of 0.84 (variance 19.6). Mean expression level of negative control probes was calculated for each tissue. The maximum mean obtained for each tissue × negative control probe was then used as a threshold level to determine *C. gigas *gene expression. According to this, 3,630 (11.4%) *C. gigas *probes had a gene expression below the background level in all tissue samples. A matrix of gene expression levels was generated, where each row corresponded to a different *C. gigas *oligonucleotide probe and each column to one tissue sample. The expression level of each contig was then logarithmically transformed, centered around 4 (the relative mean of all contigs within all tissues), and reduced so that relative variations rather than absolute values could be used for interpretation. After normalization, probes that showed gene expression at the background level had log_10 _values ranging from 2.15 to 3.15, with a mean of 2.65.

#### Microarray data analysis

We initially applied a principal component analysis (PCA) using geneANOVA software [53] to assess the internal consistency of different transcriptional data sets and to obtain the proportion of variance for each principal component. Although component 1 had the highest proportion of variance (87.4%), it did not discriminate tissue samples from each other. All samples had exactly the same proportion of variance within this component.

PC1 would represent the great majority of genes that do not show tissue-related differential expression. Components 2, 3 and 4 discriminate the tissues and were used to draw a 3D scatter plot (XLstat; Addinsoft) and to organize the 33 tissue transcriptomes along the principal components. Hierarchical clustering using Pearson's correlation was then performed using TMeV [54,55] and the results were compared to the PCA. The values of normalized signal intensities were further analyzed using one-way analysis of variance (ANOVA), with a p-value threshold of less than 0.01 and adjusted with Bonferroni correction (TMeV). Data were further filtered to determine whether their expression was specific to a tissue type. For each gene, the means of normalized expression from tissue replicates were calculated and compared to the means of normalized expression obtained for each of the other tissues.

### Quantitative RT-PCR

For quantitative RT-PCR analysis, RNA samples were treated with DNAse I (Promega; 1 U/μg total RNA) for 30 minutes at 37°C according to the manufacturer's instructions. After DNAse I treatment, absence of genomic DNA was confirmed by qPCR on a total RNA sample. Reverse transcription was then carried out using 1 μg of total RNA from each sample, 1 μg of random hexanucleotidic primers (Promega), 0.5 mM dNTPs and 200 U M-MLV reverse transcriptase (Promega) at 37°C for 1 hour in the appropriate buffer. Amplification reactions were performed in 1 × Absolute blue qPCR SYBRGreen fluorescein mix (Thermo scientific) with 5 ng of cDNA template and 300 nM of each primer in a final volume of 15 μL using an iCycler iQ™ thermocycler (Bio-Rad). The comparative threshold cycle (C_T_) method was used to quantify copy number of the target gene in the tissues. Primer sequences used in this study are listed in additional file 3. The PCR amplification efficiency (E; E = 10^(-1/slope)^) for each primer pair was determined by linear regression analysis of a dilution series [56]. The specificity of the primer pairs was confirmed by melting curve analysis at the end of each qPCR run and each amplicon was verified by gel electrophoresis. For selected tissue-specific genes, the ΔCt was calculated using *arf1 *(*adp-ribosylation factor 1*) as a reference gene and compared to the normalized expression values from the microarray analysis.

### Reference genes

Genes expressed in all tissues with a mean normalized intensity that exceeded 4 (the mean of all normalized expression values) were considered as potential reference genes for quantitative RT-PCR. One-way ANOVA with a p-value threshold < 0.05 was then performed and candidate reference genes were defined as genes that were non-differentially expressed. For each gene, the coefficient of variation (CV, the ratio of the standard deviation to the mean) was calculated. CV was used to compare the degree of variation between the genes with different means of expression [57], and the Maximum Fold Change (MFC, ratio of the maximum and minimum values) was used to reflect the minor variations of those candidates within all biological samples [58]. Genes were thus ranked in order of increasing CV to identify the top most stable candidates.

Quantitative RT-PCR was performed to identify the best candidate control genes. The Ct values were determined at a specific threshold (fixed at 500 RLU). Ct values were normalized across samples by calculating the ratio of the Ct value to the mean of the Ct value obtained for all genes for a given sample and logarithmically transformed (base 10). The CV was calculated for each gene using normalized Ct values. Normfinder [59] was used on raw and normalized Ct values to measure stability values and identify the best housekeeping gene candidates. Finally, we used geNorm on raw Ct values to determine the expression stability (M value) [60]. Results obtained from the different methods were compared to validate the selection of ideal reference genes for comparative studies among tissues.

## Abbreviations

RT-PCR: reverse transcription polymerase chain reaction; cDNA: complementary DNA; aRNA: amplified RNA; Ct: Cycle threshold; PCA: principal component analysis; PC: principal component; ANOVA: Analysis of variance; stdDev: standard deviation; M: gene expression stability measure; E: PCR amplification efficiency.

## Authors' contributions

NMD was responsible for the study design, all experimental work and analyses, and was the primary author of the manuscript. CL designed the microarray and was involved in the study design and manuscript revision. AH organized the sequence assembly in relation with Sigenae to prepare the microarray design and was involved in critically revising the manuscript. PF participated in the study design and in revising the manuscript. All authors read and approved the final version of the manuscript.

## Supplementary Material

Additional file 1 **List of 34 duplicated contigs and the gene expression values obtained within each tissue**. This table provides for each probe the sequence: Probe sequence, Row: Row on array, Col: Column on array, ProbeName, ContigName: Sigenae accession number, GB_ACC: GenBank accession number, Description: Description from Gigas Database, Duplicate probes: GenBank accession number of corresponding duplicated probe, GI: Gills; G: gonad; DG: Digestive gland; M: Mantle; MS: Adductor muscle; P: Labial palps; GG: Visceral ganglia; HE: Hemocytes, R^2^: Coefficient of determination. Click here for file

Additional file 2 **List of 200 putative genes constituted of at least 2 different contigs that show extremely similar gene expression profiles**. This table provides for each gene the ID genbank: accession number, Description from GigasDatabase, GeneName: gene name as cited in the text, mean: mean log normalized gene expression, stdev: standard deviation of log normalized gene expression, CV: coefficient of variation, max: maximum value, Min: minimum value, MFC: maximum fold change, mean R^2^: mean Coefficient of determination. Click here for file

Additional file 3 **Housekeeping genes and tissue-enriched contigs selected for quantitative RT-PCR assay**. This table provides for each contig the spot: location on microarray, ID genbank: accession number, length of primers (nucleotides), seq: 5' to 3' sequence of primer. Click here for file

Additional file 4 **Comparison of gene expression profiles of tissue-enriched contigs measured by microarray and quantitative RT-PCR analysis**. Strong homologies were observed with a coefficient of correlation (R^2^) ranging from 65 to 93%. Letters identify the tissue (GI: Gills; G: gonad; DG: Digestive gland; M: Mantle; MS: Adductor muscle; P: Labial palps; GG: Visceral ganglia; HE: Hemocytes). Click here for file

Additional file 5 **Lists of tissue-enriched contigs: male gonad, female gonad, gills, labial palps, digestive gland, mantle, adductor muscle, visceral ganglion, hemocytes**. This table provides for each contig the spot: location on microarray, ID genbank: accession number, Description from GigasDatabase, GeneName: gene name as cited in the text, mean: mean log normalized gene expression, stdev: standard deviation of log normalized gene expression, GI: Gills; G: gonad; DG: Digestive gland; M: Mantle; MS: Adductor muscle; P: Labial palps; GG: Visceral ganglia; HE: Hemocytes, SS: sum of squares, df: degrees of freedom, F ratio: ratio of the model mean square to the error mean square, adj p value: adjusted Bonferroni's correction p value. Click here for file

Additional file 6 **Lists of contigs gonad-enriched, gills- and labial palps-enriched, and ganglion- and hemocyte-enriched**. This table provides for each contig the spot: location on microarray, ID genbank: accession number, Description from GigasDatabase, GeneName: gene name as cited in the text, mean: mean log normalized gene expression, stdev: standard deviation of log normalized gene expression, GI: Gills; G: gonad; DG: Digestive gland; M: Mantle; MS: Adductior muscle; P: Labial palps; GG: Visceral ganglia; HE: Hemocytes, SS: sum of squares, df: degrees of freedom, F ratio: ratio of the model mean square to the error mean square, adj p value: adjusted Bonferroni's correction p value. Click here for file

Additional file 7 **125 candidate reference genes identified within this study**. This table provides for each gene the spot: location on microarray, ID genbank: accession number, Stdev: standard deviation, CV: coefficient of variation, max: maximum value, min: minimum value, MFC: maximum fold change. Click here for file

Additional file 8 **RNA integrity**. Provides the method employed to measure RNA integrity in oyster samples. Click here for file

Additional file 9 **GigasDatabase version 6**. All sequences of GigasDatabase version 6 in Fasta format. Click here for file
