# Supplementary material for: Development of a Pacific oyster (Crassostrea gigas) 31,918-feature microarray: identification of reference genes and tissue-enriched expression patterns
Source: BMC Genomics. 2011 Sep 27;12:468. doi: 10.1186/1471-2164-12-468 (PMC3191543; doi:10.1186/1471-2164-12-468)
Supplement: Additional file 4 — Comparison of gene expression profiles of tissue-enriched contigs measured by microarray and quantitative RT-PCR analysis. Strong homologies were observed with a coefficient of correlation (R2) ranging from 65 to 93%. Letters identify the tissue (GI: Gills; G: gonad; DG: Digestive gland; M: Mantle; MS: Adductor muscle; P: Labial palps; GG: Visceral ganglia; HE: Hemocytes). [file 1471-2164-12-468-S4.PPT]

## Slide 1
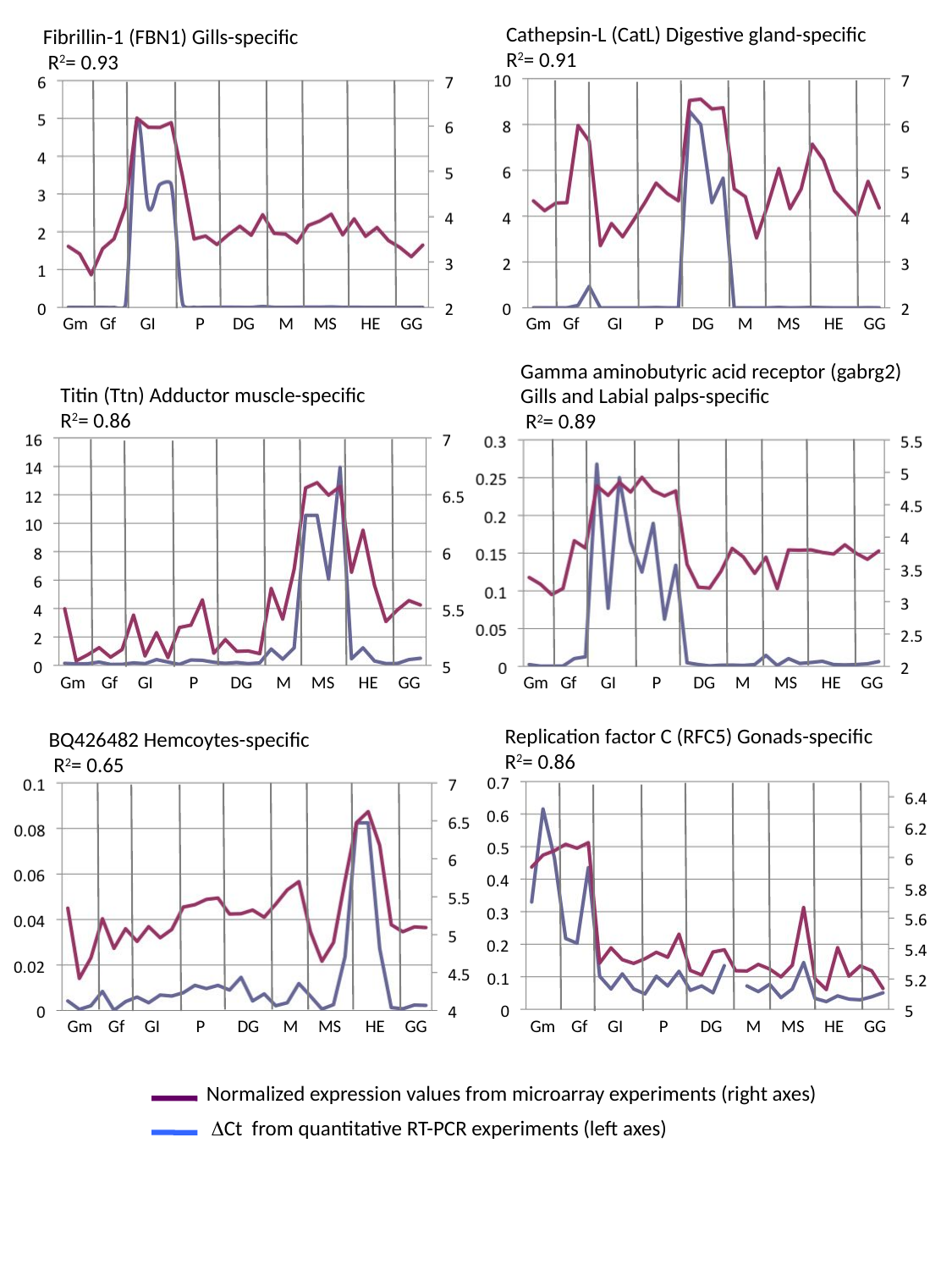

Cathepsin-L (CatL) Digestive gland-specific
R2= 0.91
Fibrillin-1 (FBN1) Gills-specific
 R2= 0.93
Gm Gf GI P DG M MS HE GG
Gm Gf GI P DG M MS HE GG
Gamma aminobutyric acid receptor (gabrg2) Gills and Labial palps-specific
 R2= 0.89
Titin (Ttn) Adductor muscle-specific
R2= 0.86
Gm Gf GI P DG M MS HE GG
Gm Gf GI P DG M MS HE GG
Replication factor C (RFC5) Gonads-specific
R2= 0.86
BQ426482 Hemcoytes-specific
 R2= 0.65
Gm Gf GI P DG M MS HE GG
Gm Gf GI P DG M MS HE GG
Normalized expression values from microarray experiments (right axes)
 ΔCt from quantitative RT-PCR experiments (left axes)
