# Supplementary material for: Development of a Pacific oyster (Crassostrea gigas) 31,918-feature microarray: identification of reference genes and tissue-enriched expression patterns
Source: BMC Genomics. 2011 Sep 27;12:468. doi: 10.1186/1471-2164-12-468 (PMC3191543; doi:10.1186/1471-2164-12-468)
Supplement: Additional file 8 — RNA integrity. Provides the method employed to measure RNA integrity in oyster samples. [file 1471-2164-12-468-S8.DOCX]

**RNA integrity control with the Agilent Bioanalyzer using RNA 6000 Nano kits**

The Agilent Bioanalyzer allows estimating the integrity of RNA samples and the ratio of 28S:18S ribosomal RNA. The automated standardized method for RNA quality control provided by the software relies on the calculation of an RNA integrity number (RIN). The RIN calculation employs numerous features, including the 18S and 28S fragment regions height, area, and intercept of the baseline (Schroeder et al., 2006)

In oysters, the 28S fragment was rarely observed and was mostly undetected by the Bioanalyzer. As documented in a range of invertebrates (Ishikawa 1977; Barcia et al., 1997; Muttray et al., 2008; Winnebeck et al., 2009), 28S absence is due to a breaking point in the rRNA structure which converts 28S into 2 fragments that are hydrogen bounded and that migrate at the same size as the 18S rRNA during gel electrophoresis.

Therefore we visually assessed the total RNA quality as demonstrated in the figure below.

Figure 1: Visual assessment of oyster total RNA quality using the Agilent Bioanalyzer. The Figure shows typical representatives of the good quality total RNA employed for microarray analyses (A and B) and the poor quality RNA that were discarded (C and D). M indicates the marker peak. 18S designs the 18S RNA fragment peak. 28S designs the remaining 28S fragment peak.

References:

Barcia R, Lopez-Garcia JM, Ramos-Martinez JI. The 28S fraction of rRNA in molluscs displays electrophoretic behaviour different from that of mammal cells. Biochemistry and Molecular Biology International 1997, 42(6): 1089-1092

Ishikawa H. Comparative studies on the thermal stability of animal ribosomal RNA’s – V. tentaculata (phoronids, moss-animals and lamp-shells). Comparative Biochemistry and Physiology Part B: Comparative Biochemistry 1977, 57(1): 9-14

Muttray AF, Schulte PM, Baldwin SA**.** Invertebrate p53-like mRNA isoforms are differentially expressed in mussel haemic neoplasia. Marine environmental research 2008, 66(4): 412-421

Schroeder A, Mueller O, Stocker S, Salowsky R, Leiber M, Gassmann M, Lightfoot S, Menzel W, Granzow M, Ragg T. The RIN: an RNA integrity number for assigning integrity values to RNA measurements. BMC Molecular Biology 2006, 7: 3

Winnebeck EA, Millar CD, Warman GR. Why does Insect RNA look degraded? Journal of Insect Science 2009, 10: 1536-2442
